# Targeting Autophagy in Innate Immune Cells: Angel or Demon During Infection and Vaccination?

**DOI:** 10.3389/fimmu.2020.00460

**Published:** 2020-03-19

**Authors:** Sha Tao, Ingo Drexler

**Affiliations:** Institute for Virology, Düsseldorf University Hospital, Heinrich-Heine-University, Düsseldorf, Germany

**Keywords:** autophagy, dendritic cells, macrophages, innate immunity, adaptive immunity, vaccines and therapies against infectious diseases

## Abstract

Innate immune cells are the “doorkeepers” in the immune system and are important for the initiation of protective vaccine responses against infection. Being an essential regulatory component of the immune system in these cells, autophagy not only mediates pathogen clearance and cytokine production, but also balances the immune response by preventing harmful overreaction. Interestingly, recent literature indicates that autophagy is positively or negatively regulating the innate immune response in a cell type-specific manner. Moreover, autophagy serves as a bridge between innate and adaptive immunity. It is involved in antigen presentation by delivering pathogen compounds to B and T cells, which is important for effective immune protection. Upon infection, autophagy can also be hijacked by some pathogens for replication or evade host immune responses. As a result, autophagy seems like a double-edged sword to the immune response, strongly depending on the cell types involved and infection models used. In this review, the dual role of autophagy in regulating the immune system will be highlighted in various infection models with particular focus on dendritic cells, monocytes/macrophages and neutrophils. Targeting autophagy in these cells as for therapeutic application or prophylactic vaccination will be discussed considering both roles of autophagy, the “angel” enhancing innate immune responses, antigen presentation, pathogen clearance and dampening inflammation or the “demon” enabling viral replication and degrading innate immune components. A better understanding of this dual potential will help to utilize autophagy in innate immune cells in order to optimize vaccines or treatments against infectious diseases.

## Introduction

The awarding of the Nobel Prize in 2016 to Yoshinori Ohsumi reflects the importance of autophagy in human health and disease. Autophagy is a homeostatic degradation process that enables cells to survive in case of stress, like accumulation of misfolded proteins and damaged organelles or starvation and energy deprivation. Mammalian cells deliver those “unwanted” materials to lysosomes for degradation. Three major ways can be distinguished: microautophagy, chaperone-mediated autophagy, and macroautophagy. The latter has been intensively characterized in recent years because of its high impact on human health and disease (1). In this review, we will focus on macroautophagy, simply referred to as autophagy in the following.

**Figure 1:**
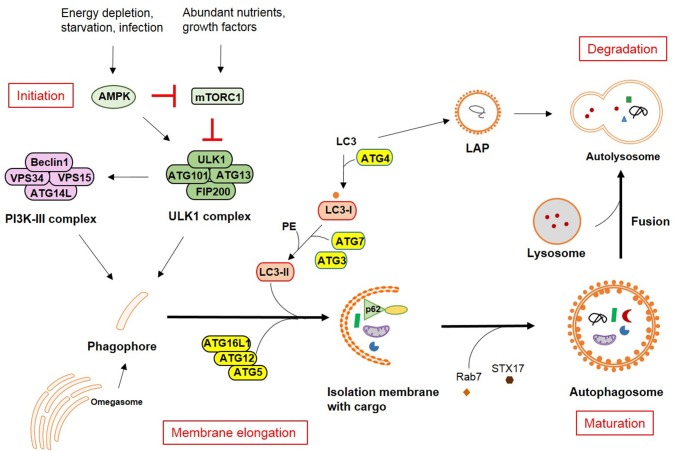
Autophagy pathways in mammalian cells. The molecular pathway comprised of the core autophagy-related proteins (ATGs) is illustrated together with the respective associated autophagy membrane compartments. The four major steps in the autophagolysosomal pathway are indicated in red.

The autophagic pathway has been widely discussed and reviewed (1–3). Here, only a brief summary will be provided, including the four major steps in the pathway: initiation, membrane elongation, maturation/fusion and degradation. In mammalian cells, after a strong stimulus such as starvation, autophagosomes initiate as omegasomes at the endoplasmic reticulum (ER). Autophagy can be induced by two different arms of upstream signaling based on either mammalian target of rapamycin (mTOR) inactivation or adenosine monophosphate (AMP) activated protein kinase (AMPK) activation which leads to distinct Unc-51-like autophagy activating kinase 1 (ULK1) activation. mTOR typically responds to nutrient signals while AMPK responds to the energy status of the cell. Two protein complexes are important for phagophore formation: ULK and PI3K (phosphoinositide 3-kinase catalytic subunit type III) complex. The ULK complex consists of autophagy-related (ATG)13, FIP200, ATG101, and ULK1. The PI3K complex comprises Beclin1, VPS34 (vacuolar protein sorting 34), VPS15 and ATG14L. Furthermore, two ubiqutin-like (UBL) conjugation complexes are important for the membrane extension. One is ATG16L1 complex, in which ATG12 is conjugated to ATG5 and then bind to ATG16L1. This facilitates another ubiquitin cascade involving ATG7 and ATG3, namely microtubule-associated protein 1A/1B-light chain 3 (LC3/ATG8) lipidation (conjugation to phosphatidylethanolamine-PE). LC3-PE mediates membrane tethering and fusion to extend the isolation membrane by recruiting membranes from multiple sources, leading to the formation of autophagosomes. During the final maturation, autophagosomes are decorated with Rab7 and tail-anchored SNAP receptor (SNARE) syntaxin 17 (STX17), which leads to the fusion with lysosomes and degradation of sequestered substrates (Figure 1). Recent findings suggest that autophagy can also occur in the absence of some key autophagy-related proteins (ATGs) through unconventional autophagy pathways, also called “non-canonical autophagy” (4–6). Furthermore, the double membrane does not necessarily elongate from a single source. Such variation gives alternatives to recognize or eliminate pathogens, for instance, receptor mediate internalization and LC3-associated phagocytosis (LAP).

The immune system is a big network with crosstalk of cells from innate and adaptive immunity. Autophagy is a key mechanism against invading bacteria, parasites, and viruses in innate immune cells including monocytes/macrophages, dendritic cells (conventional dendritic cells-cDCs and plasmacytoid dendritic cells-pDCs) and neutrophils. In the past few years, a number of studies have highlighted the potential of targeting autophagy for the control of infections. These data combined with the emerging role of autophagy for immune impairment in some infectious diseases have attracted significant interest in developing autophagy modulators or targets as a new approach for vaccination.

## Vaccines Targeting Autophagy for Effective Antigen Processing and Presentation

**Figure 2:**
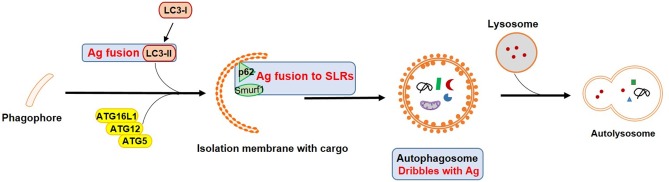
Vaccines targeting autophagy for enhanced antigen processing and presentation. Blue squares mark important autophagy components that could be targets in APCs for vaccine design by enhancing antigen presentation and T cell activation. Ag refers to antigen.

DCs as one of most potent professional antigen-presenting cells (APCs) bridge between the innate and adaptive immune system. They are particularly critical for naïve T cell activation and drive protective immunity against infection. An increasing number of recent studies have characterized the involvement of autophagy in various DC functions in physiological as well as pathological context (7), especially with regard to T cell activation (8, 9) (Figure 2).

Autophagy has been shown to be involved in antigen processing and presentation in DCs, especially for MHC class II restricted peptides. Those peptides are often derived from lysosomal degradation, either from LAP (non-canonical pathway) (10) or from macroautophagy (canonical pathway). In the latter, cytosolic antigens are recognized by a group of SQSTM1-like receptors (SLRs) such as p62, NDP52, OPTN or NBR1. This selective form of autophagy is also called “xenophagy” (3, 11). SLRs serve as links to ubiquitin (tagged with substrates) and LC3 homologs on the autophagosomal membrane. Peptides are further loaded on MHC class II in late endosomal MHC class II containing compartments (MIIC) (12). LAP involves single-membraned phagosomes, but also leads to MHC class II presentation (5). As a result, it is not surprising that autophagy promoted MHC class II antigen presentation to CD4+ T cells in various infection models, such as modified vaccinia virus Ankara (13) or herpes simplex virus 1 (HSV-1) (14–17) or was able to enhance cytokine production by CD4+ T cells in *Toxoplasma gondii* (*T. gondii*) (18) or respiratory syncytial virus (RSV) infection (19). Indeed, enabling access of antigens to autophagolysosomes by genetic engineering to link them to key components like LC3-II, greatly enhanced vaccine efficacy. For instance, human immunodeficiency virus-1 (HIV-1) Gag and Env fail to colocalize with LC3 containing vesicles during infection. However, once antigens were targeted to LC3b, the autophagic degradation process was enhanced and could efficiently stimulate CD4+ T cell responses (20, 21). Besides, autophagy also indirectly promoted antigen presentation by benefitting lysosomal enzyme activity during HIV-1 infection (22). Further examples include the conjugation of influenza A virus (IAV) matrix protein 1 (M1) to LC3 in DCs which led to enhanced antigen-specific CD4+ T cell responses (23). Japanese encephalitis (JEV) prM and E proteins fused to LC3 (pJME-LC3 DNA vaccine) allowed for increased T cell responses and long lasting antibody-mediated protection after immunization (24).

In addition to MHC class II presentation to CD4+ T cells, autophagy in several APCs has been considered to contribute to MHC class I presentation to CD8+ T cells. Indeed, pDCs (25) as well as some subtypes of macrophages (26) showed potential for antigen capture and processing, and promoted T cell priming in infection models. Viral peptides derived from autophagosomes were further processed by proteasomes in HIV-1 infected macrophages (27). The MHC class I presentation of human cytomegalovirus (HCMV) protein pUL138-derived peptide epitopes was autophagy-mediated and TAP-independent (28). H1N1 infected bone marrow-derived DCs (BMDCs) were activating strong CD4+ T cell proliferation and additionally, were more efficiently cross-presenting antigen to CD8+ T cells (29). All these reports suggest an interaction between vacuolar and MHC class I presentation pathways. Dribbles are autophagosomal structures derived from tumor cells after proteasomal inhibition and are currently tested as tools to enhance cross-presentation. Human DCs loaded with DRibbles isolated from tumor cells expressing CMV peptide epitopes were significantly more efficient in stimulating CD8+ memory T cells (30). Similarly, DCs loaded with DRibbles containing CMV proteins revealed a superior ability to induce CMV-specific T cells (31).

Among SLRs, p62 and NDP52 are considered most important for pathogen recognition through autophagy. p62 delivers ubiquitinated *Mycobacterium tuberculosis* (Mtb) proteins into autolysosomes for clearance (32, 33), while clearance of Bacillus anthracis is based on rapid induction of LC3 conversion, Beclin1 expression and p62-mediated degradation in neutrophils (34). Redirection of vaccine antigens from proteasomal degradation into autophagosomal pathways could increase the generation and variability of antigen-specific T cells. Fusion of HIV-1 Gagp24 to the selective autophagy receptor sequestosome 1 (SQSTM1)/p62 complex enhanced antigen delivery and increased antigen-specific T cell responses in comparison to Gagp24 alone (35). The connection of p62 and autophagy is highly conserved between species and could be an interesting candidate for T-cell-based vaccine strategies in humans. More recently, another recognition molecule in selective autophagy captured attention regarding autophagy-mediated host defense against infection. Smurf1 is an E3 ubiquitin ligase and a key component in autophagic targeting of Mtb in macrophages supporting host defense *in vivo* (36) which may suggest a new potential target for enhancing xenophagic degradation.

Recently, a self-assembling peptide vaccine in which the amphipathic peptide KFE8 (FKFEFKFE) was either combined with MHC class II restricted epitopes from Mtb Ag85B or MHC class I restricted peptides from ovalbumin. These conjugate vaccines were tested *in vitro* in APCs with known ability to induce strong antibody and cellular responses to conjugated antigens. Interestingly, both variations were processed through autophagy and displayed a highly efficient antigen presentation capacity to T cells (37). However, the vaccine efficacy still needs to be established *in vivo* and for other target antigens.

## Adjuvants That Enhance Vaccine Efficiency Through Autophagy

**Figure 3:**
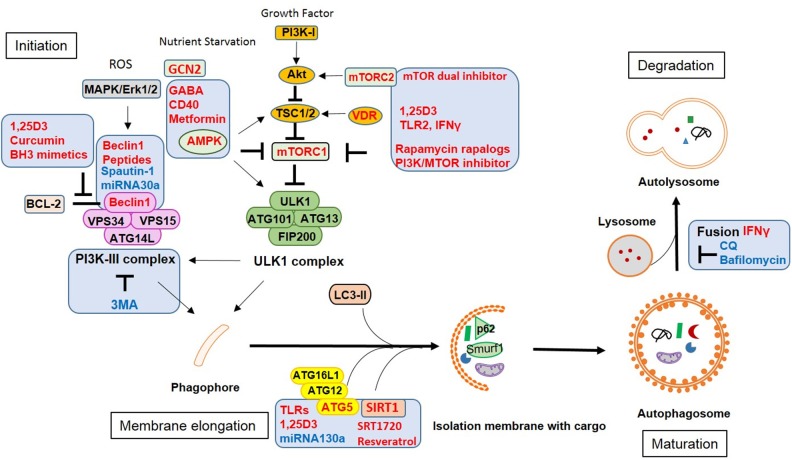
Vaccine adjuvants and therapeutic strategies against infection by modulating autophagy. Approaches or targets aiming to enhance autophagy are labeled in red, those inhibiting autophagic functions are labeled in blue.

Some vaccines are derived from attenuated strains of pathogens. Deleting virulence genes increases the vaccine safety but sometimes also reduces immunogenicity, especially when the lost genes are associated with autophagy functions. In order to enhance vaccine efficacy, the boosting of host immune responses with adjuvants which induce autophagy may increase phagocytosis and clearance of pathogens as well as antigen presentation by innate immune cells (Figure 3).

For instance, Bacillus Calmette-Guerin (BCG) representing a live attenuated strain from *Mycobacterium tuberculosis* (Mtb) is still used as a vaccine for tuberculosis (TB). However, it's efficiency varies and is especially low in adults. Therefore, vaccine adjuvants have gained great interest to improve BCG vaccines. Compared to Mtb, the attenuated BCG lacks a functional ESX-1 system (secreting ESAT-6 and CFP-10). This system allows cytosolic components of ubiquitin-mediated autophagy to access phagosomes and to free contained mycobacteria which supports bacterial evasion from xenophagic elimination (38) and reduces antigen presentation (39). Combination of BCG vaccines with autophagy inducers or with peptides from the above mentioned virulence proteins showed better protection *in vivo* than BCG alone. A BCG vaccine that overexpressed immunogenic Ag85B has been proven superior compared to the wild-type BCG vaccine. Particularly, additional application of rapamycin enhanced Ag85B-specific MHC class II presentation by DCs via autophagy and thus increased vaccine efficacy (40). An autophagy inducing and TLR2 activating C5 peptide from Mtb-derived CFP-10 protein was overexpressed in BCG in combination with Ag85B (BCG^85C5^). This recombinant BCG^85C5^ induced robust LC3-dependent autophagy in macrophages which increased antigen presentation to CD4+ T cells *in vitro* and enhanced effector and central memory T cell responses *in vivo* (39). Accordingly, a recombinant BCG ΔureC::hly(+) (rBCG) vaccine with enhanced AIM2 inflammasome activation and autophagy was more efficient against TB in preclinical animal models than parental BCG (41). Consequently, triggering other synergistic innate pathways in conjunction with autophagy should boost the immune response and vaccine efficacy.

DCs play a key role for antigen presentation and T cell induction during viral infections. Autophagy has been shown to be involved in DC maturation during RSV infection (42) which enabled DCs to migrate to secondary lymphoid tissues, interact with naïve T cells and trigger effector T cell responses (43). HIV-1 protein Env has been proposed to downregulate autophagy by activating mTOR and reducing lysosomal enzyme activity in DCs, thereby inhibiting antigen presentation and enhancing the transfer of infection into CD4+ T cells (22, 44). However, viral inhibition was bypassed by rapamycin-mediated stimulation of autophagy in DCs and restored CD4+ T cell responses (44). Starvation is an efficient way to induce autophagy. By activating the cellular starvation sensor general control non-derepressible 2 (GCN2) kinase, yellow fever vaccine YF-17D induced autophagy in human DCs which operated via the induction of cell death in infected “donor” cells and the enhanced uptake by bystander DCs through LAP, which subsequently resulted in enhanced antigen presentation and robust T cell responses (45). Beclin1, one important component of canonical autophagy also proved to act as a potent vaccine adjuvant. By enhancing autophagy-mediated antigen presentation, a combinatory HPV-16 E7/Beclin1 DNA vaccine led to superior lymphocyte proliferation and cytotoxicity as compared to the HPV-16 E7 DNA vaccine alone (46).

Additional to traditional adjuvants inducing autophagy such as rapamycin, several drugs may be repurposed from their original usage (e.g., against cancer or diabetes), since they activate autophagy and increase vaccine-specific immune responses. For instance, curcumin is known for inducing autophagy in tumor cells and is currently tested for tumor therapy. Due to its poor intestinal absorption and rapid metabolism, this drug has clinical limitations. However, a recent paper demonstrated that a nanoparticle-formulated version of curcumin could overcome these limitations and optimized various APC functions including autophagy and finally enhanced BCG vaccine efficacy by inducing central memory T cell as well as Th1 and Th17 responses (47). Another biological, the antioxidant glutathione (GSH), improved BCG vaccination by increasing autophagy and the production of IFNγ and TNFα which prevented loss of T cells by decreasing the expression of PD-1 (48). However, those adjuvants have not been widely used in other infection models and the effect could be pathogen-specific.

## Therapeutic Approaches Against Infections by Modulating Autophagy

Since autophagy has important functions in innate and adaptive immunity, it is not surprising that pathogens employ autophagy to escape the host immune response. In macrophages, the Gram-positive bacteria *Listeria monocytogenes* (Lm), mycobacteria (Mtb) including BCG, and parasites such as *Toxoplasma gondii* (*T. gondii*) are well-studied intracellular pathogens that have different strategies to utilize autophagy for long-term survival or replication (49), while LAP (5) as well as xenophagy (11) usually result in bacterial degradation by lysosome fusion. However, Lm masks its surface with ActA and InlK to avoid recognition (50, 51), while Mtb evades killing by CpsA which prevents a robust oxidative response (ROS) and further leads to LAP (52). To bypass these defects, treatment of innate immune cells with autophagy inducers could enhance pathogen killing (Figure 3). Common therapies include adenosine monophosphate–activated protein kinase (AMPK) modulators. CD40 may stimulate autophagic killing of *T. gondii* in macrophages via calcium/calmodulin-dependent kinase kinase ß (CaMKKß), AMPK and ULK1 (53) and subsequent fusion of vacuoles and late lysosomes (54). Gamma-aminobutyric acid (GABA) or GABAergic drugs promote autophagy via intracellular calcium release and AMPK signaling resulting in enhanced phagosomal maturation and subsequently antimicrobial responses against mycobacterial infection (55). The U.S. Food and Drug Administration (FDA)-approved antidiabetic drug metformin increases the production of mitochondrial reactive oxygen species (ROS) and facilitates phagosome-lysosome fusion which has been shown to limit Mtb growth and to reduce chronic inflammation in infected mice (56).

Another option to enhance autophagy is through vitamin D receptor (VDR) activation and cathelicidin induction. The active form of vitamin D, 1,25-dihydroxyvitamin D3 (1,25D3), induces autophagy in human monocytes and activates transcription of the autophagy-related genes Beclin1 and ATG5 (57). A combination of retinoic acid (RA) and vitamin D3 (VD) (RAVD) enhanced the levels of DC-SIGN and mannose receptors on THP-1 macrophages which increased mycobacterial uptake and inhibited the subsequent intracellular growth of Mtb by inducing ROS and autophagy (58). TLR2/1/CD14 stimulation activates antibacterial autophagy through VDR and cathelicidin in human primary monocytes (59). Additionally, some effector cytokines such as IFNγ activate autophagy in macrophages. IFNγ treated macrophages could overcome the inhibition of phagosome-lysosome fusion by Mtb and controlled intracellular Mtb growth (60). In severely ill TB patients, IFNγ as well as Th1 and Th17 immune responses are required to eliminate Mtb. Interestingly, IL17a alone was unable to augment autophagy because of a disease-associated defect in MAPK1/3 signaling. Adding IFNγ to IL17a increased autophagy levels in the patients' monocytes resulting in strong immunity to Mtb and promoting mycobacterial killing (61).

Though autophagy plays a key role in macrophages and neutrophils for pathogen clearance through xenophagy or LAP, it is still unclear how phagosomes trigger autophagy. One paper suggests a connection to TLR signaling, but this has not been investigated in infectious context (62). Autophagy may not always work best to eliminate pathogens. Previous reports have shown that phagocytosis of Escherichia coli triggers the autophagic machinery in neutrophils (63). On the other hand, autophagy may reduce the phagocytosis rate of mycobacteria in murine macrophages (64) and neutrophils (65). The reduced internalization is due to decreased expression of two class A scavenger receptors, namely macrophage receptor with collagenous structure (MARCO) and macrophage scavenger receptor 1 (MSR1) (66). Therefore, pharmacological modulation of autophagy should aim to target both, autophagy and phagocytosis, and should be carefully designed for each pathogen.

Clearing blood-borne pathogens is a hallmark feature of the spleen. In this secondary lymphoid organ, pDCs and red pulp macrophages efficiently cleared malaria parasites after boosting autophagy with rapamycin (67, 68). The treatment enhanced antigen presentation in these cells and shifted the cytokine and chemokines profile *in vivo* which recruited effector cells into the spleen and enhanced T cell responses. Thus, targeting autophagy in pDCs and red pulp macrophages may open new prospects for the development of novel antimalarial drugs.

When it comes to viral infection, some viral proteins directly inhibit autophagy. For instance, HIV-1 Nef is interacting with Beclin1 which blocks the late stage of autophagy in macrophages, thereby protecting virus particles from degradation (69). That is able to suppress IFNγ-induced autophagy in infected macrophages (70) as well as in bystander macrophages through Src-Akt and STAT3 signaling (71). Rescue of autophagy function could be a novel approach to prevent and treat HIV-1 infection and related opportunistic infections. The PI3K/MTOR inhibitor (dactolisib) and PI3K/MTOR/BRD4 inhibitor (SF2523, JQ1) restrain HIV replication through degradative autophagy without altering the initial susceptibility of macrophages to infection (72). Low vitamin D levels in HIV-1 infected patients are associated with more rapid disease progression and increased risk for other infections such as Mtb. 1,25D3 targets multiple steps in autophagy and inhibits HIV replication and mycobacterial growth in co-infected human macrophages through the induction of autophagy (73). In RSV infection, Sirtuin 1 (SIRT1) an NAD+ dependent deacetylase regulates autophagy depending on the nutrient status. SIRT1 may induce autophagy directly by deacetylating TAG5 and 7 and LC3. Activated DCs produce crucial cytokines promoting antiviral Th1 responses, while pathologic Th2 and Th17 responses are suppressed during infection (74). SIRT1 inducers like SRT1720 could be a therapeutic alternative for RSV patients.

Autophagy may have distinct functions depending on the stage of infection or the host cell type infected (75, 76). In the early phase of HSV-1 infection, autophagy was found to be transiently induced in human THP-1 cells favoring viral replication (77). In the later phase, however, viral protein ICP34.5 blocked the maturation of autophagosomes which reduced viral antigen presentation by DCs (17). Alternatively, human gamma herpesviruses EBV and KSHV regulate autophagy in immune cells during *de novo* infection, while autophagy plays a distinct role in chronic murine gamma herpesvirus 68 (MHV-68) infection by triggering virus reactivation from latency (78). Thus, opposing effects of the same drug may occur depending on the infection settings. While rapamycin induces killing of Mtb in macrophages, it supports Mtb growth during low-dose and controlled infection when co-infected with HIV-1 by interfering with phagosomal maturation (35). Therefore, a detailed knowledge of how pathogens modulate autophagy during the infection cycle will help to develop more specific targets for autophagy-based strategies against infectious diseases.

Various viral virulence factors such as ICP34.5 (HSV-1), Nef (HIV-1), or M11 (MHV-68) have been shown to specifically interact with autophagy-related proteins such as Beclin1 to exploit autophagy for viral replication. New therapy approaches that target specific components of autophagy pathways, which are manipulated by pathogens, should maximize clinical benefits while minimizing toxicity. For instance, an autophagy inducing cell-permeable peptide (Tat–Beclin1) has been generated by mapping the functional region of Beclin1 with the HIV-1 Tat transduction domain (PTD). Therapeutic application of Tat-Beclin1 was associated with reduced HIV-1 replication, decreased intracellular survival of the bacterium Lm in human macrophages and reduced mortality of mice infected with chikungunya or West Nile virus (79). Inhibitors of interactions between viral and autophagic proteins may also have potential benefits for the prevention and treatment of a broad range of human diseases. Bcl-2 binds to Beclin1 preventing assembly of pre-autophagosomal structures which inhibits autophagy. This interaction involves a Bcl-2 homology 3 (BH3) domain in Beclin1. Proteins containing BH3 domains such as BH3 mimetics (80, 81) can competitively disrupt the interaction between Beclin1 and Bcl-2 and thereby induce autophagy (82). Another example is MHV-68 M11 which binds to one BH3 domain of Beclin1 and inhibits autophagy. Alternatively, a Beclin1 BH3 domain-derived peptide which selectively binds to M11, but not to Bcl-2, abrogated M11-mediated down-regulation of autophagy (83) (Figure 3).

Autophagy is additionally regulated by mitochondrial integrity and contributes to the elimination of damaged organelles. Autophagy may selectively degrade inflammasome components such as NLRP3 and products such as Pro-IL1ß (84) thereby limiting the secretion of pro-inflammatory cytokines. As a result, the dampening of inflammation is another important role of autophagy in innate immune cells which so far has been exclusively shown in myeloid cells. For instance, loss of autophagy-related proteins in macrophages causes massive inflammation after infection which may lead to host tissue damage such as lung injury in IAV (85, 86) and Mtb infection (87) as well as in pseudomonas aeruginosa-driven abdominal infection (88). Autophagy also helps to curtail virus-induced systemic inflammation by creating an environment that prevents host injury mediated by pro-inflammatory cytokines. Trichostatin A (TSA), an autophagy inducer used to reduce systemic inflammation and to attenuate sepsis-induced organ injury, promotes M2 polarization in peritoneal macrophages and ultimately improved the survival of mice with polymicrobial sepsis (89). In some autoimmune diseases such as inflammatory bowel disease (IBD), the containment of inflammation may prevent the development of disease and reduce the risk of infections. Enhancing autophagy may be therapeutically beneficial by regulating inflammation and clearing intestinal pathogens. Recently, IL-10 was reported to induce mitophagy (autophagy-mediated mitochondria degradation) to prevent accumulation of dysfunctional mitochondria and production of mitochondrial ROS in macrophages from IBD patients (90) (Figure 3).

## Therapeutic Strategies Aiming to Inhibit Autophagy to Block Virus Replication and Immune Evasion

Autophagy seems to be an angel for most infection scenarios, except for two: first, autophagy may benefit virus replication rather than support host immune protection as seen for flaviviruses. Second, autophagy may degrade components of innate immune pathways which negatively affects innate sensing of pathogens and antiviral cytokine production. The former represents a potential therapeutic target for several (re-)emerging diseases for which we currently have no vaccine available due to rapid spread and high virulence. The latter is exemplified by multiple targets in the cGAS/STING DNA sensing pathway aiming to halt IFN production. A direct interaction between cGAS and Beclin1 has been described in macrophages during HSV-1 infection (91). Similarly, p62-mediated degradation of cGAS in HSV-1 and VSV infection (92) as well as poxin-mediated cGAMP degradation by various poxviruses has been reported (93). The latter has not been associated with autophagy so far, but the therapeutic potential of any autophagy modulator that may arise from these studies has not been assed and molecular mechanisms still need to be elucidated.

Some viruses induce autophagy for their own replication by taking advantage of membrane structures produced in this process as reported for flaviviruses [hepatitis C virus (HCV), dengue virus (DENV), zika virus (ZIKV)] or hepatitis B virus (HBV) (94). For viruses that infect specific organs or tissues like HBV or HCV, tissue-specific targeting of autophagy e.g., hepatocytes, would be favorable. HCV genomic RNA is recognized by RIG-I, MDA5 and TLRs to activate IFN signaling and pro-inflammatory cytokine secretion. HCV induces autophagy in hepatocytes which enables its replication and trafficking, additionally, attenuates the innate immune response by viral proteins NS3 and NS5A (95). Consequently, knockdown of autophagy-related proteins (Beclin1 or ATG7) in immortalized human hepatocytes (IHH) could inhibit HCV growth (96) and block exosome-mediated virus transmission (97).

For DENV and ZIKV, some autophagy pathway components are crucial for replication including maturation and packaging. The induction of autophagy by ZIKV appears to be linked to the activation of AMPK, while DENV induces autophagy by activation of VPS34 (98). USP10 and USP13 are needed to reverse ubiquitination and subsequently degradation of the Beclin1-VPS34-ATG14 complex. Targeting this complex by Spautin1 which inhibits the deubiquitination activity of the two molecules inhibited DENV infection (99). In innate immune cells, the pro-inflammatory cytokine macrophage migration inhibitory factor (MIF) induces autophagy and facilitates DENV replication. Inhibition of MIF-induced autophagy by minocycline might represent an alternative therapeutic approach against DENV infection (100). Pharmacological inhibitors of autophagolysosomal activity such as chloroquine (CQ) prevents endosomal viral RNA release and autophagy-dependent viral replication and is currently used to prevent maternal to fetal transmission of ZIKV (101–103) (Figure 3).

MicroRNAs (miRNAs) represent a new tool to regulate autophagy by specifically targeting the expression of autophagy-related genes. They bind to the target mRNA through specific base-pairing interactions between the “seed” region of miRNA and sites within coding and untranslated regions (UTRs) of mRNAs, especially 3′UTRs, to suppress gene expression. Several miRNAs have been shown to augment or repress virus replication through interfering with autophagy. Therefore, manipulation of cellular miRNAs which target autophagy components represent a novel approach for purging pathogens. Administration of miRNA130a diminished HCV replication by interfering with ATG5-dependent autophagy (104), while miRNA146a targets TRAF6 which blocked DENV-induced autophagy in THP-1 cells (105). Enterovirus 71 (EV71)-induced autophagy is mediated by Beclin1 which contains a potential binding site for miRNA30a. By using a miRNA30a mimic, EV71 replication was suppressed by blocking virus-induced autophagy (106). This therapeutic approach is widely used in the tumor field, but the options for different pathogens may vary, since miRNA expression is altered during conditions of stress and disease (Figure 3).

## Future Prospects

Innate immune cells are the first line of defense against infection. Targeting autophagy in those cells is an attractive approach to augment vaccination efficacy or to improve immunotherapeutic strategies against infectious diseases. Vaccines which are based on genetic fusion of antigens to important components of autophagy pathways improved adaptive immune responses by enhancing antigen processing and presentation in APCs. Combination with autophagy modulators as adjuvants has been proven to further boost host immune responses by triggering innate immunity as well as increasing immune cell functions such as cytokine/chemokine production, maturation, and migration. Pharmacological induction of autophagy could increase pathogen clearance in phagocytes through xenophagy or LAP. Furthermore, given the potent anti-inflammatory effect associated with autophagy, employing autophagic functions in myeloid cells might also help to control infections in some autoimmune diseases. The functions or effector mechanisms exerted by or related to autophagy during infection may vary among cell types, the type of pathogen or the stage of infection. The impact of autophagy goes beyond single cell types and involves intensive cross-talk within the whole immune system and therapeutic strategies may have to be determined individually for a given pathogen. Future studies will have to focus on investigating the role of autophagy for pathogen–host-specific interplay *in vivo* and identify relevant steps in the course of infection in which the targeting of autophagy—possibly in selected cell types—proves to be most efficient for pathogen clearance and protection. This will allow to develop new strategies for vaccines or therapeutic approaches with optimized efficacy against infectious diseases and help to minimize unwanted off-target effects or toxicity.

## Author Contributions

ST and ID wrote the manuscript. ST generated images.

### Conflict of Interest

The authors declare that the research was conducted in the absence of any commercial or financial relationships that could be construed as a potential conflict of interest.
